# Chloramine Catch: Water Disinfectant Can Raise Lead Exposure

**Published:** 2007-02

**Authors:** John Tibbetts

Many water treatment systems around the nation have stopped using chlorine to disinfect drinking water. Chlorine reacts with dissolved organic matter in water to create by-products that are suspected of causing human health problems, including some forms of cancer. Many water treatment plants now use disinfectants called chloramines, combinations of chlorine and ammonia. But in some water systems this switch has coincided with an increase in lead in drinking water, perhaps because chloramines cause lead to leach from pipes, fixtures, and solder. Now a team of researchers from Duke University has measured the potential effect of switching from chlorine to chloramines on blood lead levels **[*EHP* 115:221–225; Miranda et al.]**.

The scientists used geographic information system–based software to link blood lead data, housing data (dissolved lead in water can occur only when a lead source is present, a condition that is much more likely in older housing), drinking water sources, and census data for 7,270 children in Wayne County, North Carolina. Blood lead data were obtained from a statewide registry of all blood lead screens conducted on North Carolina children under the age of six. The authors noted that the lead-screened children were well distributed across different ages of housing in Wayne County.

The county has two main public water systems. About 70% of the residential tax parcels get drinking water through Wayne Water Systems, which uses chlorine for disinfection. Another 28% of parcels get drinking water through the Goldsboro Water System, which has used chloramines for disinfection since March 2000.

The Goldsboro Water System’s change to chloramines was associated with an increase in children’s blood lead levels, suggesting that use of chloramines could lead to an increase in lead exposure. The impact of the change to chloramines was progressively mitigated in newer housing, however. In houses built after 1950, the newness of the home was a stronger influence on blood lead than the use of chloramines.

Much uncertainty still surrounds the underlying environmental chemistry of how combinations of disinfectants, anticorrosives, coagulants, and fluoridation agents combine with water qualities such as pH, alkalinity, temperature, oxidation potential, and concentrations of other chemical species to affect lead in drinking water. Nevertheless, these results provide guidance to both water systems and health departments on which houses should be targeted for monitoring of lead in both water and residents’ blood.

## Figures and Tables

**Figure:**
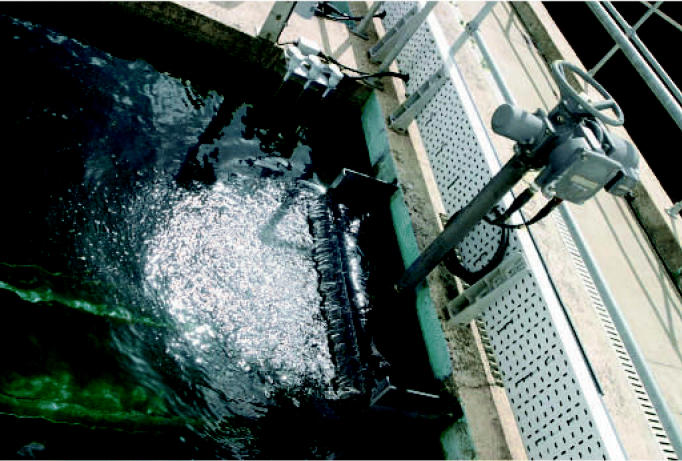
Trading effects? Chloramines do not create toxic by-products like chlorination but may increase residents’ lead exposures.

